# Evaluating Changes in Physical Activity and Clinical Outcomes During Post-Hospitalisation Rehabilitation for Persons with COPD: A Prospective Observational Cohort Study

**DOI:** 10.3390/s26020384

**Published:** 2026-01-07

**Authors:** Anna L. Stoustrup, Phillip K. Sperling, Lars P. Thomsen, Thorvaldur S. Palsson, Kristina K. Christensen, Jane Andreasen, Ulla M. Weinreich

**Affiliations:** 1 Respiratory Research Aalborg, Department of Respiratory Diseases, Aalborg University Hospital, 9000 Aalborg, Denmark; 2 Department of Clinical Medicine, The Faculty of Medicine, Aalborg University, 9000 Aalborg, Denmark; 3 Department of Anaesthesiology and Intensive Care, Aalborg University Hospital, 9000 Aalborg, Denmark; 4 Respiratory and Critical Care Group, Department of Health Science and Technology, The Faculty of Medicine, Aalborg University, 9000 Aalborg, Denmark; 5 Department of Physiotherapy and Occupational Therapy, The Faculty of Medicine, Aalborg University Hospital, 9000 Aalborg, Denmark; 6 Public Health and Epidemiology, Department of Health Science and Technology, The Faculty of Medicine, Aalborg University, 9000 Aalborg, Denmark; 7 Aalborg Health and Rehabilitation Center, Aalborg Municipality, 9000 Aalborg, Denmark

**Keywords:** COPD, pulmonary rehabilitation, physical activity, frailty, accelerometer

## Abstract

**Highlights:**

**What are the main findings?**

- Triaxial accelerometers provided detailed patterns of daily physical activity during post-hospitalisation rehabilitation after acute exacerbation of COPD.
- Despite improved self-perceived health, accelerometer-derived physical activity levels and clinical outcomes remained stable throughout the 8-week exercise program.

**What are the implications of the main findings?**

- Continuous accelerometer monitoring can complement clinical and patient-reported outcomes in evaluating rehabilitation interventions.
- Findings highlight the potential of sensor-based assessments to support personalised and long-term rehabilitation strategies after hospitalisation.

**Abstract:**

Physical activity often remains low after hospitalisation for acute exacerbation of Chronic Obstructive Pulmonary Disease (AECOPD). Although post-hospitalisation rehabilitation has shown to support recovery, its impact on daily activity levels in the early post-exacerbation phase is unclear. This study describes the changes in physical activity (PA) and clinical outcomes during an 8-week rehabilitation following hospitalisation for AECOPD. This prospective observational cohort study included patients recently discharged after AECOPD from Aalborg University Hospital, Denmark. Participants received municipality-delivered post-hospitalisation rehabilitation consisting of tailored physiotherapy and occupational therapy of individually determined frequency. PA was assessed using thigh-worn triaxial accelerometers measuring 24 h/day over 8 weeks. Clinical outcomes included lung function (FEV1% predicted), dyspnoea scores, health-related quality of life (EuroQol 5-dimension, 5-level (EQ-5D-5L); EuroQol visual analogue scale (EQ-VAS)), frailty (Clinical Frailty Scale (CFS)), functional status (Short Physical Performance Battery (SPPB)), and symptom burden (COPD Assessment Test (CAT); St. George’s Respiratory Questionnaire (SGRQ)). Changes from baseline to 8 weeks were analysed using linear mixed-effects models and bootstrap resampling. Forty-three participants with a mean age 73.4 years, 67.4% female, and moderate frailty (CFS 5.4 ± 1.3) were included. Physical activity remained largely unchanged. Gait speed and total SPPB declined, whereas self-perceived health improved (EQ-VAS Δ +7.8, *p* = 0.008). Lung function, dyspnoea, and health related quality of life scores showed no significant change. In this frail, recently admitted COPD population, physical activity did not increase during the rehabilitation period, and some functional parameters declined. The improvement in self-perceived health suggests a divergence between subjective and objective outcomes. These findings highlight the need for long-term, tailored, and flexible approaches to support recovery after AECOPD.

## 1. Introduction

Reduced physical activity in persons with Chronic Obstructive Pulmonary Disease (COPD) is associated with poorer health outcomes, including functional decline, reduced health-related quality of life (HRQoL), increased risk of hospitalisations, and higher all-cause mortality [1,2,3]. Research suggests that persons with COPD are less active than healthy individuals [4]. The decline in physical activity (PA) in persons with COPD is caused by respiratory symptoms, fatigue, muscle dysfunction, systemic inflammation, hospitalisations, and associated comorbidities related to oxidative stress [5,6,7]. Psychological barriers, including anxiety and depression, further impact PA [8,9,10,11]. Despite these challenges, engaging in regular activity is important for effective COPD management, improving pulmonary function, increasing activity, and reducing the over-all health burden [1,12,13,14]. International clinical guidelines emphasise promoting activity as a key component in rehabilitation interventions [14].

Early rehabilitation during the post-hospitalisation period could mitigate the risks of future exacerbations and promote functional recovery [14,15]. While pulmonary rehabilitation (PR) is a well-established intervention for improving physical capacity and HRQoL [12,13,14], the association of early rehabilitation post-hospitalisation on PA remains underexplored. Current guidelines recommend that patients begin PR within 3 weeks after a hospitalisation for COPD [14], and a systematic review by Puhan et al. showed significant benefits of early rehabilitation but stated that the results were heterogeneous in terms of their effect on hospitalisations and mortality [13]. Similarly, a Danish randomised controlled trial (RCT) found that early municipality-based PR initiated after two weeks led to improved physical performance compared to PR initiated after two months [15]. However, most studies focus on survival, readmissions, and functional performance.

While the importance of physical activity in individuals with COPD is well-documented, little is known about the early post-hospitalisation phase following an AECOPD, since most rehabilitation studies are performed with stable-phase persons with COPD [16]. Despite improvements in the clinical outcomes, the effects on the daily activity levels in the critical post-exacerbation phase remain unclear. No studies have examined PA using continuous thigh-worn accelerometry during the early post-hospitalisation rehabilitation period after an acute exacerbation of COPD (AECOPD). PA in patients with COPD can be assessed using subjective methods like questionnaires or diaries or device-based methods like accelerometry. Device-based assessment is often preferred when detailed behavioural patterns are of interest or when symptom burden and slow gait may limit the accuracy of self-report or binary step-based metrics [17]. This study therefore aims to address this gap by providing descriptive accelerometer data on PA patterns during acute recovery. The study also explores clinical outcomes during this period. Addressing this gap may support the development of more tailored rehabilitation strategies [18].

### Study Aim

This study aimed to evaluate the changes in physical activity levels and clinical outcomes during post-hospitalisation rehabilitation for persons with COPD following AECOPD. Specifically, we sought to (1) assess the changes in daily activity levels during acute rehabilitation and (2) evaluate the clinical outcomes before and after participation in the acute rehabilitation program.

## 2. Materials and Methods

### 2.1. Study Design

This prospective cohort study was conducted through a collaboration between Aalborg University Hospital, Denmark, and Aalborg Municipality, Denmark, from October 2023 to August 2024. It was part of the COPDtoParis Project [19], an RCT investigating the effects of long-term home-based cycling for patients recently admitted with AECOPD. This cohort study served as a prelude to the RCT, examining patterns of change in physical activity levels and clinical outcomes during the acute rehabilitation period, prior to randomisation into the trial. Aalborg Municipality provided the rehabilitation, as required by Danish law. This study was reported in accordance with the STROBE (Strengthening the Reporting of Observational Studies in Epidemiology) guidelines [20].

### 2.2. The Acute Rehabilitation Framework

The legislated acute rehabilitation program in Denmark provides structured rehabilitation services for persons who have experienced functional decline post-hospitalisation, including persons with COPD recovering from AECOPD. Mandated by law (the Danish Health Act §140) [21], the acute rehabilitation program offers government-funded rehabilitation tailored to individual needs, including physio- and occupational therapy. The service is not standardised across patients. Instead, the content, frequency, duration, and mode of delivery (centre-based or home-based) are individually determined following a clinical assessment conducted by the municipality. Typical components may include supervised exercise, functional training, mobility practice, respiratory physiotherapy, and occupational therapy [21]. This study therefore describes outcomes under real-world rehabilitation conditions rather than a controlled intervention. The inherent variability in the program delivery reflects the structure of usual care and is an important contextual factor when interpreting changes in physical activity and clinical outcomes.

### 2.3. Participants

Participants were recruited from the Emergency Department and the Department of Respiratory Diseases at Aalborg University Hospital, Denmark. Eligible participants were adults aged 18 years or older, residing in Aalborg Municipality, with a pre-existing COPD diagnosis and a recent hospitalisation due to an exacerbation. Further, the inclusion criteria were the ability to provide informed consent and being suitable for participation in the acute rehabilitation program.

The exclusion criteria included medical conditions that contraindicated participation in physical rehabilitation or an inability to understand basic oral and written Danish. Recruitment and enrolment were managed by trained physiotherapists, who daily screened patient lists from the Emergency Department and Department of Respiratory Medicine to identify eligible patients. After identification, the principal investigator provided oral and written study information [19]. Details on the participant flow, attrition, and valid measurements are presented in the Results section.

### 2.4. Primary and Secondary Outcomes

**Figure 1:**
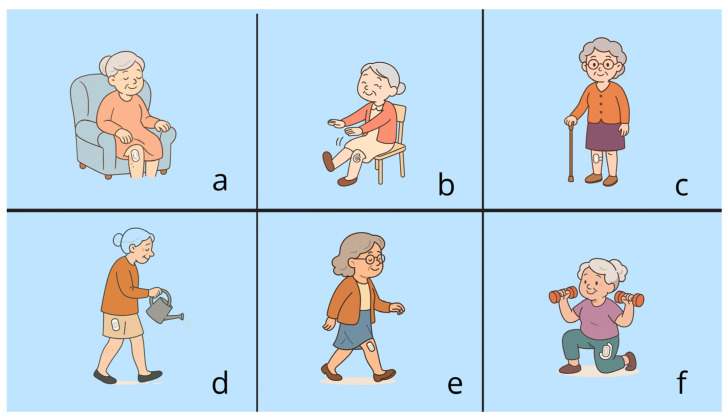
Examples of activities corresponding to the six domains derived from thigh-worn triaxial accelerometer monitoring. The categories include (**a**) sedentary (resting or sitting without movement), (**b**) sitting/lying movement (minor postural adjustments while seated or lying), (**c**) standing (upright posture without stepping), (**d**) sporadic walking (short intermittent steps during daily tasks), (**e**) continuous walking (prolonged stepping with regular gait pattern), and (**f**) moderate activity (activities producing higher movement intensity). These domains formed the basis for stratification and analysis of changes in physical activity over time.

The primary outcome of this study was the change in daily sedentary time over 8 weeks/56 days after start of acute rehabilitation. Physical activity levels were continuously measured using triaxial accelerometers (SENS^®^, SENS Innovation ApS, Copenhagen, Denmark), mounted on participants’ thighs. Activity data were categorised into sedentary time, lying/sitting movement, standing, sporadic walking, continuous walking, and moderate activity, as shown in Figure 1. Outcomes were the total time (minutes per day) spent in each activity, using a proprietary algorithm from SENS. This activity classification approach has been previously validated for recognition of posture and movement patterns in older adults and clinical populations [22,23]. The SENS^®^ system and activity classification have been evaluated in validation studies against direct observation, including in populations with a slow gait and clinical vulnerability. This supports its use for quantifying activity patterns [17,24]. Secondary outcomes included the change in other activity categories.

Additional clinical outcomes included lung function (forced expiratory volume in 1 s in percent of predicted (FEV1%)), dyspnoea measured by the Modified Medical Research Council Dyspnoea Scale (mMRC) [25], and functional status assessed using the Clinical Frailty Scale (CFS) [26]. Health-related quality of life was evaluated with the EuroQol 5-Dimension 5-Level instrument (EQ-5D-5L) and EQ-VAS [27]. Respiratory symptom burden was measured by the COPD Assessment Test (CAT) [28], and physical performance was assessed using the Short Physical Performance Battery (SPPB) [29]. Finally, disease-specific quality of life was evaluated using the St. George’s Respiratory Questionnaire (SGRQ) [30].

### 2.5. Statistical Analysis

Baseline characteristics are presented using descriptive statistics. Continuous variables are reported as the mean ± standard deviation (SD). Categorical variables are presented as frequencies and percentages. The primary outcome was analysed using a linear mixed-effects model.

Participants wore the thigh-mounted accelerometers continuously (24 h/day) for the full 8-week period. Participants were instructed to keep the sensor attached during daily activities. If temporary removal was necessary, to change the sticky patch that the sensor was placed in, participants were instructed to reattach the sensor with a clean patch as soon as possible. Since the devices were worn at all times, including sleep, non-wear thresholds were not applicable. Instead, a day was considered valid if a complete 24 h recording was measured. Days with interrupted measurements, due to device power-off or signal dropout, were excluded from weekly aggregation. Weekly aggregates were calculated as the mean of all 24 h days within that week. Participants with missing days entered the multiple imputation procedure described below. No participants were excluded due to missing days. Unlike studies aimed at estimating habitual activity from a short assessment window, e.g., 7 days, this study used 8-week monitoring to describe week-by-week activity trajectories across the acute rehabilitation period.

A linear mixed-effects model was used to account for the differences between participants. This approach was chosen to account for repeated measurements within individuals and to handle inter-individual variability in baseline levels and change trajectories, while allowing the inclusion of participants with incomplete datasets.

The activity level was the outcome (dependent variable), while the week was set as a fixed-effect predictor. The patient ID was included as a random effect to adjust for repeated measurements within individuals. “Week” was modelled as a continuous variable to capture the linear time trend across the 8-week period. To relax assumptions about linearity over time, we performed sensitivity analyses with Week entered as a categorical factor. The same modelling was used to assess the other activity categories.

Before fitting the mixed models, standard visual checks of the model residuals (histograms, Q-Q plots, and plots) were performed, to assess normality and homoscedasticity. The inclusion of a random intercept for each participant accounted for the repeated measurements over time. Residual autocorrelation was examined. Analysing all weekly observations rather than only baseline to follow-up helped reduce potential regression-to-the-mean effects.

Missing data (e.g., sensor signal loss, patient dropout, or death) were addressed with multiple imputations with chained equations (MICE). Five imputed datasets were generated using Predictive Mean Matching (PMM) with nearest-neighbour matching. The imputation model included all outcomes and baseline covariates used in the analyses. Data were suspected to be missing at random, given that the primary reason for missing sensor data was technical errors that were not related to the measured activity, e.g., running out of battery or storage capacity. Predictive mean matching was chosen to preserve the observed distribution of continuous variables and to avoid imputing implausible values. Five imputations were applied, and the results from the imputed datasets were compared with complete-case analyses as a sensitivity check. The convergence and stability of the imputations were evaluated by visual inspection of the summary statistics across iterations.

For clinical outcomes, *p*-values were estimated using a bootstrap resampling procedure. For each hypothesis test, 5000 bootstrap replicates were generated by resampling with replacement. The null distribution of the mean difference was estimated by calculating the mean difference for each resampled dataset (start of the rehabilitation minus start of intervention for each participant). A two-tailed *p*-value was calculated by counting how often the absolute mean difference in the bootstrap samples was higher than zero and then doubling this proportion. Bootstrapping served as a robustness check against deviations from normality.

The patient ID was specified as a random effect, since participants may be random samples from a broader population of persons with COPD eligible for the provided service, and our primary interest was in the average time trend rather than in subject specific estimates. Week was treated as a fixed effect, since the week was the time point of interest in this study.

To account for skewed data and the potential non-normality of residuals, bootstrapping with 5000 resamples was conducted. All analyses were conducted in Stata (version 18; StataCorp LLC, College Station, TX, USA). The analyses were conducted by an experienced statistician and validated by the study team.

### 2.6. Ethical Approval and Informed Consent

This study was approved by the North Denmark Region Committee on Health Research Ethics (N-20230008) and complies with the Helsinki Declaration. All participants provided written informed consent prior to inclusion in the study.

## 3. Results

### 3.1. Study Population

**Figure 2:**
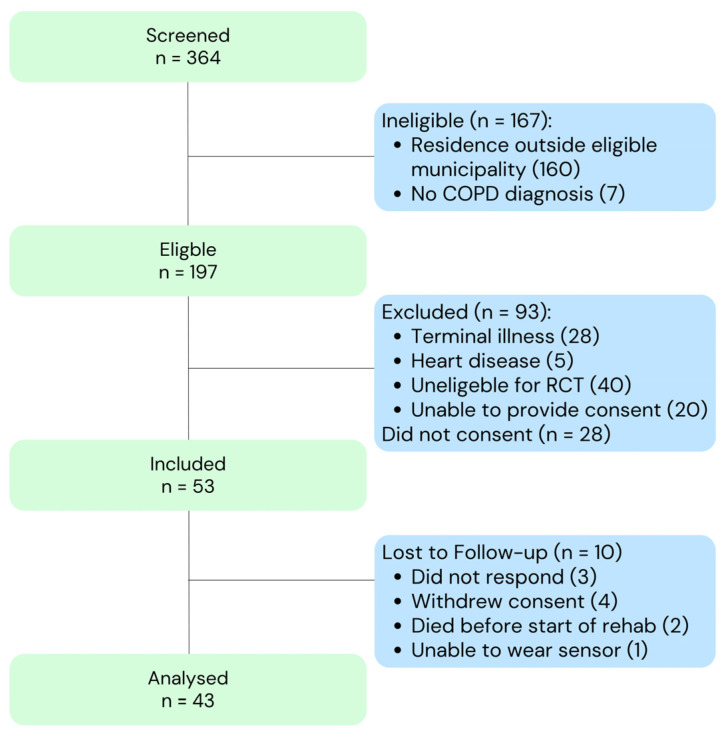
Flowchart showing screening, eligibility assessment, inclusion, exclusion, and follow-up for participants in the observational COPD cohort. A total of 364 individuals were screened, of whom 197 were eligible. After exclusion due to clinical criteria, inability to consent, or declining participation, 53 participants were included. Ten were lost to follow-up, resulting in 43 participants in the final analysis. COPD: Chronic Obstructive Pulmonary Disease; RCT: randomised controlled trial.

A total of 43 persons with COPD were included in this study. Figure 2 provides an overview of the screening, inclusion, and exclusion. During follow-up, attrition was primarily due to the clinical deterioration typical of this frail post-AECOPD population. Five participants (12%) died, and additional loss to follow-up occurred due to withdrawal or inability to wear the sensor.

The descriptive baseline statistics for the demographics and clinical characteristics are detailed in Table 1.

### 3.2. Changes in Physical Activity

Table 2 summarises the changes in physical activity levels across the rehabilitation period, analysed using a mixed-effects model. Physical activity monitoring yielded variable results. The mixed-effects model showed no statistically significant change during the 8-week period (slope: 1 min/day, 95% CI −8.2 to 10.2, *p* = 0.8). A significant decrease was observed in “movement while sitting/lying”. No other significant changes were found. Standing showed a numerical increase, while walking showed a numerical decrease. The observed changes correspond to small shifts, with a considerable inter-individual variation across all categories. This variation should be considered when interpreting the mean changes, as the clinical relevance of a given change differs depending on baseline activity level. Together, these results indicate that the sedentary time did not follow a systematic upward or downward trajectory at the group level.

### 3.3. Clinical Outcomes

Table 3 presents the changes in clinical outcomes during the rehabilitation period. Overall, the symptoms remained stable in terms of lung function, dyspnoea, and CAT scores. In contrast, self-perceived health (EQ-VAS) improved significantly. Physical function showed mixed results, where the SPPB dimensions of total, balance, and sit-to-stand remained unchanged, and the gait speed statistically significantly declined.

## 4. Discussion

### 4.1. Key Findings

This study aimed to examine changes in physical activity and clinical outcomes during an 8-week post-hospitalisation rehabilitation for persons with COPD. Our main findings were that, while self-perceived health, measured by the EQ-VAS, statistically significantly improved, the physical activity measures remained largely unchanged or even slightly declined.

Despite participating in a structured rehabilitation program, most participants did not increase their daily physical activity in any dimension. The increase in self-perceived health suggests an internal inconsistency between objective and subjective outcomes. Our findings align with previous studies, that have shown low translation from PR to increased physical performance. Egan et al. also reported no significant improvements in activity levels post-PR [31]. A study from the European COPD Audit showed that patients often do not regain their functional capacities after an exacerbation [32], supporting our observation that functional recovery is difficult to achieve. This is supported by previous studies that have shown that lower activity levels are associated with higher risks and health complications in persons with COPD [1,33].

It is notable that the participants in our study had a low baseline activity and were moderately frail, indicating limitations in independence and daily function. This could partly explain the limited physical capacity to sustain or improve more active activity patterns. Güell et al. [34] did, however, observe improvements in both the short term and long term, although in a setting where participants received a three-year maintenance exercise program, suggesting that this group might benefit from long-term interventions. These differences may also be due to varying demographics, as well as the duration, intensity, and support of the intervention.

The coexistence of stable PA measurements and the decline in gait speed should be interpreted cautiously. PA and functional performance represent related but distinct constructs [35]. Changes in performance may reflect other contributors than the rehabilitation. This study is observational, and hence, the findings cannot be attributed solely to the content or delivery of the rehabilitation program. The municipal rehabilitation provided under the Danish Health Act §140 varies in delivery. As such, our results describe a real-world setting with multi-facetted recovery trajectories rather than impact of an intervention. Importantly, the exercises, intensity, and frequency of the municipal rehabilitation were individually determined and not recorded in this study. Consequently, the period between the baseline and follow-up is not characterised in detail. This limits the interpretation and translation of the findings of this study.

Mortality, loss of data, and lost to follow-up reflect the clinical vulnerability of this cohort [36]. Missing days were due to death or technical interruptions rather than non-adherence, and the similarity between the complete-case and imputed data estimates suggests that these patterns did not materially change the findings. However, since the participants were frail, and five died during follow-up, we cannot exclude selection bias. Those who survived and provided full datasets may represent a subgroup with higher resilience.

Compared to Kjaergaard et al. [15], who found improvements in walking capacity with the similar acute rehabilitation program in Denmark in a similar cohort comparing early PR to delayed initiation of PR, our population was frailer and perhaps had reduced reserve capacity for functional improvements. While Kjaergaard et al. saw functional improvements, it did not translate to readmissions or survival long-term [15]. This suggests that short-term rehabilitation programs may produce limited functional improvements, and the effects of long-term physical activity remain to be explored.

The observed discrepancy between the largely unchanged activity patterns, symptoms, and physical performance and the significant improvement in self-perceived health, may reflect the complexity of recovery after AECOPD. Participants might have experienced meaningful improvements in emotional well-being or daily coping. These were not dimensions measured in this study. This could suggest these dimensions as relevant measurements, as they might reflect what is important to persons during rehabilitation. PA and functional capacity are related yet distinct outcomes. The decline in gait speed may reflect limited physiological reserve, frailty, or natural disease progression during this phase rather than an effect of the rehabilitation program. The discrepancy could also suggest the importance on supporting transfer from exercise to everyday activity through, e.g., behavioural support, activity planning, and strategies to reduce sedentary time.

Several factors may contribute to the largely unchanged PA and physical outcomes. First, the low baseline activity and degree of frailty may limit the exercise reserve and the ability to translate exercise sessions into higher PA. Second, this cohort was small and in a vulnerable post-AECOPD phase with substantial morbidity, attrition, and competing demands, which may exceed short-term gains. These findings should not be interpreted as evidence against rehabilitation in the acute post-AECOPD phase but rather as indicating that short-term trajectories may be insufficient for some frail patients with COPD after hospitalisation.

While acute rehabilitation after AECOPD may support early recovery for many, the current setup or time frame might not be sufficient, especially for persons with significant frailty, more sedentary lifestyles, or persons nearing the end-of-life stage. The large interpersonal variation observed in physical activity behaviour and clinical outcomes points toward the need for more stratified and personalised approaches in rehabilitation [18,37]. This could help identify who would benefit from more intensive programs. Others may require other interventions than intense exercising. This is supported by previous research indicating that baseline capacity and frailty status influence responses to rehabilitation for persons with COPD post-hospitalisation [38,39,40,41]. Future interventions may benefit from integrating flexible and personalised rehabilitation approaches, including longer follow-up, a higher degree of support, and low-threshold activities, like home-based exercise [42,43], to improve adherence and promote sustained increases in physical activity.

The availability and accessibility, as well as the quality and quantity, of PR vary across settings, partly due to geographical, organisational, and economic factors. This could potentially influence patient outcomes [44]. Moreover, many persons with COPD do not access the available PR despite the benefits [2]. These studies highlight the need for high quality accessible rehabilitation interventions to ensure better patient outcomes. In Denmark, the accessibility, quality, and quantity vary considerably, which may lead to gaps in care quality [45,46]. Studies underscore the need for a high quality of care and the standardisation of rehabilitation programs to ensure even access and quality in rehabilitation.

Considering the study design, variability in rehabilitation delivery, and uncertainty related to selection and missing data, conclusions about clinical relevance or implementation pathways should be made carefully. Instead, our findings highlight the challenges of capturing improvements in real-world municipal rehabilitation and the importance of exploring individualised, longer-term, or differently structured approaches.

### 4.2. Strengths and Limitations

A strength of this study is the objective measurement of physical activity using triaxial accelerometers. These reduce the recall bias associated with self-reported activity measures, as well as more precisely measuring the increased gait variability of these persons with COPD [47,48]. Stratifying the activity into six different dimensions is, furthermore, a strength, as binary outcomes such as pedometers might not measure the participant standing more. Additionally, the prospective cohort design allows for the evaluation of natural changes in physical activity and clinical outcomes in a real-life setting, since these patients would have been eligible for referral for acute rehabilitation, regardless of participating in the RCT. The study was conducted within a structured rehabilitation framework, reflecting usual clinical practice.

Several limitations should be acknowledged. First, the content and delivery of the rehabilitation was provided by the municipality, variations in the program structure, duration, and intensity across participants were not recorded, and the exercise dose could therefore not be quantified. The specific nature of the intervention delivered is likely linked with the outcomes and the patients’ status and capacity. Second, adherence to rehabilitation was not recorded, and varying levels of participation may have influenced the findings [49,50]. Information on pharmacological treatment was not collected; therefore, the potential influence of medication use, including agents with possible musculoskeletal effects, could not be evaluated. Third, the study duration was limited to 8 weeks. Long-term effects could not be assessed due to the subsequent RCT intervention in this population. Future research should evaluate the long-term effects of acute pulmonary rehabilitation independently. In this part of our study, objective measurements were used, while qualitative aspects such as participant motivation, barriers, and psychological factors were not assessed. These could have provided deeper insights into the observed activity patterns. Finally, this was a study with a small sample size, which limits the generalisability of our findings.

## 5. Conclusions

While the self-perceived quality of life improved during the 8-week rehabilitation period, objective measures of physical activity levels and functional capacity remained largely unchanged. These findings provide a descriptive insight into real-world recovery after AECOPD and illustrate the complexity of linking rehabilitation participation with behavioural or functional change. Because the rehabilitation delivery was not standardised or fully characterised, the results cannot be interpreted as evidence of program effect but rather highlight the variability and vulnerability that shape recovery trajectories in this population.

## Figures and Tables

**Table 1:** Demographics and baseline characteristics.

Characteristics		Summary
Age		73.4 ± 9.4
Female (%)		29 (67.4)
FEV1% Predicted		44.1 ± 11.2
BMI, kg*m^−2^		25.4 ± 7.3
Pack-years		45.6 ± 19.9
Clinical Frailty Scale		5.4 ± 1.3
Smoking n (%)		
	Former Smokers	34 (81.0)
	Current Smokers	8 (19.0)

Categorical variables are presented in absolute numbers and continuous variables as the mean and standard deviation (SD). FEV1: forced expiratory volume. BMI: body mass index.

**Table 2:** Changes in physical activity (with imputed data).

Activity Measure	Week 1 (min/day)	Week 8 (min/day)	Difference	*p*-Value	Slope Estimate (min/day) [95% Confidence Interval]
Sedentary (min/day)	1204.9	1212.1	7.1	0.8	1 [−8.2–10.2]
Lying/Sitting Move (min/day)	22.7	18.3	−4.4	0.03	−0.6 [−1.2–−0.1]
Standing (min/day)	96.9	114.3	17.3	0.5	2.5 [−4.1–9.1]
Sporadic Walking (min/day)	64.7	54.1	−10.7	0.2	−1.5 [−3.6–0.6]
Continuous Walking (min/day)	48.0	38.7	−9.3	0.1	−1.3 [−3–0.4]
Moderate Activity (min/day)	0.6	0.5	−0.1	0.8	0 [−0.1–0.1]

Data based on triaxial accelerometer output; baseline defined as mean of week 1 and follow-up as mean of week 8. Data are reported as mean changes in minutes per day. Statistical significance indicates within-subject changes from baseline to follow-up.

**Table 3:** Clinical outcomes from baseline to 8 weeks of rehabilitation.

Variable		n	Baseline	Post-Rehabilitation	Mean Difference, [95% CI]	*p*-Value
FEV1% Predicted		37	44.6 ± 11.1	44.9 ± 11.2	0.2 [−2.1–2.3]	0.814
mMRC Dyspnoea Score		36	2.7 ± 1.3	2.6 ± 1.2	0 [−0.3–0.2]	0.930
EQ-5D-5L		35	0.6 ± 0.2	0.7 ± 0.2	0.1 [0–0.2]	0.040
EQ-VAS		35	52.6 ± 15.2	60.4 ± 16.1	7.8 [2–14.1]	0.008
CAT Score		34	17.7 ± 8.1	16.8 ± 8.6	−1 [−2.5–0.6]	0.229
SPPB						
	Balance	35	3.0 ± 1.1	3.1 ± 1.2	0.1 [−0.2–0.5]	0.514
	Gait speed	34	2.6 ± 0.9	1.4 ± 0.9	−1.2 [−1.6–−0.9]	<0.001
	Chair Stand Test	22	2.8 ± 1.2	2.7 ± 1.2	−0.1 [−0.6–0.4]	0.804
	Total	35	7.4 ± 3.1	6.3 ± 3.3	−1.1 [−2–−0.3]	0.017
SGRQ						
	Symptoms	35	58.4 ± 14.2	60.3 ± 15.0	1.9 [−2–5.6]	0.322
	Activity	35	71.6 ± 13.9	77.9 ± 14.1	6.3 [1.8–11.6]	0.004
	Impact	35	38.3 ± 12.9	39.9 ± 13.1	1.6 [−3.4–6.7]	0.530
	Total	35	53.1 ± 14.9	55.7 ± 15.0	2.6 [−0.9–6.5]	0.145

Baseline and post-rehabilitation values are reported as the mean and standard deviation. Mean differences are reported with 95% confidence intervals [CI]. CI = 95% confidence interval. FEV1 = forced expiratory volume in 1 s; mMRC = modified Medical Research Council dyspnoea scale; EQ-5D-5L = EuroQol 5-dimension, 5-level; EQ-VAS = EuroQol visual analogue scale; CAT = COPD Assessment Test; SPPB = Short Physical Performance Battery; SGRQ = St. George’s Respiratory Questionnaire.

## Data Availability

In accordance with the ICMJE recommendations, individual de-identified participant data will be made available upon reasonable request. This includes study protocols and statistical analysis plans. These data will be available for as long as the current infrastructure stands. Access to these data will be granted to researchers who provide a methodologically sound proposal for use in achieving the aims outlined in the approved proposal. Proposals should be directed to the corresponding author.

## Abbreviations

The following abbreviations are used in this manuscript:

COPD	Chronic Obstructive Pulmonary Disease
AECOPD	Acute Exacerbation of Chronic Obstructive Pulmonary Disease
EQ-5D-5L	EuroQol 5-Dimension 5-Level
EQ-VAS	EuroQol Visual Analogue Scale
SPPB	Short Physical Performance Battery
CAT	COPD Assessment Test
SGRQ	St. George’s Respiratory Questionnaire
HRQoL	Health-Related Quality of Life
PR	Pulmonary Rehabilitation
RCT	Randomised Controlled Trial
STROBE	Strengthening the Reporting of Observational Studies in Epidemiology
FEV1	Forced Expiratory Volume in one second
mMRC	Modified Medical Research Council Dyspnoea Scale
SD	Standard Deviation
MICE	Multiple Imputation by Chained Equations
PMM	Predictive Mean Matching
